# *Announcement*: National Stroke Awareness Month — May 2017

**DOI:** 10.15585/mmwr.mm6618a8

**Published:** 2017-05-12

May is National Stroke Awareness Month, an observance that highlights the importance of knowing the signs and symptoms of stroke and encourages persons to act FAST (Face drooping, Arm weakness, Speech difficulty, Time to call 9–1–1) if someone is having a stroke. Stroke is the fifth leading cause of death in the United States and a leading cause of severe disability (*1*,*2*). In the United States, one person dies from stroke approximately every 4 minutes (*2*).

Stroke is preventable and largely treatable. Yet, a recent CDC report notes that the age-adjusted death rate for stroke slightly increased from 36.5 deaths per 100,000 persons in the United States in 2014 to 37.6 in 2015 (*1*). Approximately 60% of persons who die from stroke are women, and women tend to have worse functional outcomes after experiencing a stroke (*3*). CDC urges everyone to learn the warning signs of stroke and take action to reduce their risk. Living a healthy lifestyle (e.g., being physically active, eating more fruits and vegetables and foods low in sodium and salt, maintaining a healthy weight, and avoiding smoking) can reduce the chances of having a stroke. Properly managing certain medical conditions (e.g., high blood pressure, high cholesterol, heart disease, and diabetes) also can lower the risk.

CDC promotes stroke prevention through several initiatives. The Million Hearts initiative, co-led by CDC and the Centers for Medicare & Medicaid Services, works to prevent stroke. The Paul Coverdell National Acute Stroke Program (https://www.cdc.gov/dhdsp/programs/stroke_registry.htm) funds state health departments to collect and use data to ensure high-quality, statewide systems of care to treat stroke. Additional information regarding stroke prevention is available at https://www.cdc.gov/stroke/.
